# Facial pain and multiple cranial palsies in a patient with skin cancer

**DOI:** 10.1007/s10194-011-0324-6

**Published:** 2011-03-31

**Authors:** Janina Viken, Lars Bendtsen, Klaus Hansen, Morten Katholm, Henning Laursen, Nina Hastrup, Peter Gideon, Messoud Ashina

**Affiliations:** 1 Department of Neurology, Glostrup Hospital, 2600 Glostrup, Denmark; 2 Danish Headache Center and Department of Neurology, Glostrup Hospital, 2600 Glostrup, Denmark; 3 Department of Neurology Rigshospitalet, University of Copenhagen, 2100 Copenhagen, Denmark; 4 Department of Otolaryngology Rigshospitalet, University of Copenhagen, 2100 Copenhagen, Denmark; 5 Neuropatholgy Laboratory Rigshospitalet, University of Copenhagen, 2100 Copenhagen, Denmark; 6 Department of Neuroradiology Rigshospitalet, University of Copenhagen, 2100 Copenhagen, Denmark

## Introduction

Perineural tumor invasion is a rare complication of cancer though well-reported phenomenon among patients with head and neck cancer. Multiple cranial neuropathies as an initial symptom of recurrent neoplasm have been reported in few studies. The latest study by Leach et al. [1] reported multiple cranial nerve involvement in 67% (4 out of 6 patients) of the patient in comparison to 21% (13 out of 62 patients) found in a study of Mendenhall et al. [2]. Facial pain, progressive weakness of the facial nerve and involvement of fifth cranial nerve were the symptoms most often referred by the patients in previous studies [1–5]. The similarities of the symptoms with Bell’s palsy, trigeminal neuralgia or facial pain of uncertain etiology can lead to misdiagnosis and postpone treatment of highly morbid tumors. Here we describe a patient with multiple cranial neuropathies due to perineural spread of squamous cell carcinoma (SCC), whom the diagnostic procedure establishment of a final diagnosis was a long and challenging process because of repetitive non-diagnostic biopsies and negative magnetic resonance imaging (MRI).

## Case report

**Fig. 1:**
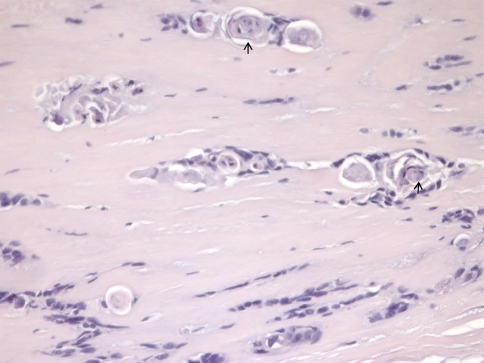
Infraorbital nerve: severely fibrotic endoneurium infiltrated with planocellular carcinoma (*arrows*) (H&E × 400)

**Fig. 2:**
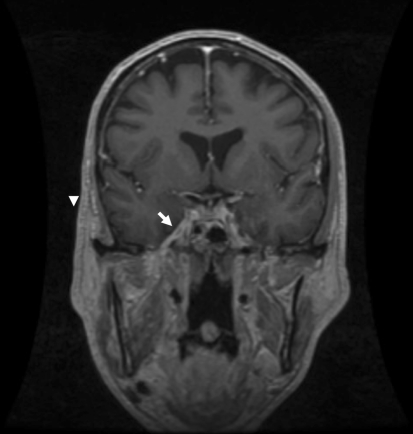
MRI coronal view shows perineural enhancement of the right maxillary nerve (*arrow*), and enhancement around cavernous sinus and the Meckel cave

In January 2005, a 78-year-old male was referred from general practice to the Danish Headache Center for investigation and treatment of right-sided facial pain. He had a recurrent cancer in his right buccal region, and received curettage and electrodesiccation in August 2001, in January 2002, and in October 2002. Each time skin biopsies were obtained. The first and second biopsy showed basal cell carcinoma (BCC), and the third suggested SCC. He was subsequently examined twice (March 2003 and September 2004) by a dermatologist and declared clinically cured. In May 2003, he developed a white spot, numbness, and burning pain in the right buccal region. In September 2004, he complained of increased pain and experienced pain after non-painful stimuli (allodynia) in the region supplied by the maxillary branch of the trigeminal nerve. In October 2004, he developed incomplete right-sided facial nerve palsy (decreased nasolabial fold). In February 2005, MRI scans with focus on the fifth and seventh cranial nerves and cerebrospinal fluid (CSF) examinations were unremarkable. The patient complained of constant burning and intermittent stabbing facial pain with average intensity 7 (0–10 pain intensity numeric rating scale). He was treated with gabapentin (3,600 mg daily), codeine (125 mg daily), amitriptyline (50 mg daily) and ketobemidone (125 mg daily as need), but shortly after stopped with all medications because of adverse events. Two months later, he still complained of severe facial pain and developed diplopia. The patient was placed on pregabalin (450 mg daily) and reported a decrease in pain intensity by 40–50%. In May 2005, a neurological examination revealed unilateral (right sided) third and sixth cranial nerve palsies, complete loss of sensory function of the right trigeminal nerve (V1, 2, 3), temporal muscle atrophy, and ninth cranial nerve palsy. The perineural spread of his cutaneous cancer was suspected. A MRI scan showed no signs of perineural spread, and CSF and whole body positron emission tomography examinations were normal. A biopsy of the lingual nerve was unremarkable and a biopsy of the right supraorbital nerve showed axonal degeneration and subtotal loss of the myelinated nerve fiber, but no signs of perineural spread. A biopsy of the infraorbital nerve revealed infiltration of both blood vessels and nerve by SCC (Fig. 1). In July 2005, MRI scans showed pathological perineural enhancement of the right maxillary nerve and around the cavernous sinus and the Meckel cave (Fig. 2). A re-evaluation of the skin biopsies from 2001 to 2002 revealed SCC. The patient underwent radiation therapy without adjuvant chemotherapy over a period of two months. On a follow**-**up examination, 2 months after start of radiation, the neurological status and mild facial pain were unchanged. Three months later, the patient died of pneumonia.

## Discussion

Nonmelanoma skin cancers are the most common forms of cancer. BCC accounts for nearly two-thirds of skin cancers whereas SCC accounts for 10% of skin cancers. Perineural spread of nonmelanotic skin cancer is still not well recognized. The literature reports an incidence of perineural spread of 1% for BCC and 2–14% for SCC [6–8]. Patients usually present with paresthesia, followed by neuropathic pain in the area previously affected by the skin cancer. The trigeminal nerve, in particular the maxillary division (40% of cases), and the facial cranial nerve are most commonly involved [6–11]. Once within the cranial cavity, the perineural spread may disseminate rapidly and involve the third, fourth and sixth nerves causing diplopia [5, 6]. Partial ipsilateral cranial neuropathies may progress over several months. When a patient with previous skin cancer in the face develops ipsilateral trigeminal or facial neuropathy perineural spread should be suspected. Although careful history and clinical assessment are essential diagnostic tools, establishment of a final diagnosis of perineural spread may be a long and challenging process. Physicians should be aware of the diagnostic pitfalls involved. In the early course of perineural spread of cancer, MRI may be unremarkable and multiple nerve biopsies may be necessary before a diagnosis is confirmed. Our case also emphasizes the importance of follow-up and early detection of perineural spread and treatment to prevent spread in the cranial cavity.
